# Intraoral lipomas: Review of literature and report of two clinical cases

**DOI:** 10.4317/jced.52926

**Published:** 2016-12-01

**Authors:** Sonia Egido-Moreno, Ana-Belén Lozano-Porras, Siddharth Mishra, Marcos Allegue-Allegue, Antonio Marí-Roig, José López-López

**Affiliations:** 1 DDS. Dentist, Master of Oral Medicine, Surgery and Implantology / Professor of the Master of Oral Medicine, Surgery and Implantology. University of Barcelona, Spain; 2 DDS. Dentist, Student of Master of Oral Medicine, Surgery and Implantology, University of Barcelona, Catalonia, Spain; 3 DDS. Dentist, Professor of the Master of Oral Medicine, Surgery and Implantology, University of Barcelona, Catalonia, Spain; 4 PhD, MD, DDS. Professor of the Master of Oral Medicine, Surgery and Implantology, Faculty of Dentistry, University of Barcelona. Specialist in Maxillofacial Surgery, Head of Department of Maxillofacial Surgery at University Hospital of Bellvitge. Catalonia, Spain. Oral health and masticatory system Group (Institute of Biomedical Research of Bellvitge) IDIBELL, University of Barcelona, Catalonia, Spain; 5 PhD, MD, DDS, Professor of the Master of Oral Medicine, Surgery and Implantology. Department of Odontostomatology, University of Barcelona. Professor of Oral Medicine, Faculty of Dentistry, University of Barcelona. University of Barcelona, Spain / Chief Medical Surgical Service of Dental Hospital University of Barcelona / Oral health and masticatory system group (Institute of Biomedical Research of Bellvitge) IDIBELL

## Abstract

**Background:**

Lipomas are benign mesenchymal tumors composed of mature adipocytes. They are classified according to their histological pattern and their etiology remains unclear. Objectives: To present two cases and review the literature.

**Material and Methods:**

A search was conducted in the Medline / PubMed and Scielo data bases of the last 10 years (2004-2014) with the keywords “ intraoral lipoma OR oral cavity lipoma”.

**Results:**

46 articles with 95 cases (56 women and 39 men) were reviewed. The average age was found to be 52.28 years (52.28 ± 18.55); and most of them occurred between the 4th and 6th decade of life. Lipomas occur mostly in the buccal mucosa (n = 36, 37.9%), followed by the tongue (n = 23, 24.2%) and other locations (n = 36, 37.9%). The most common histologic pattern was simple lipomas (n = 40, 42%), followed by fibrolipomas (n = 18, 18.9%) and other types (n = 37, 39.1%). The average tumor size was 19.77 ± 16.26mm.

**Conclusions:**

Lipomas are a relatively rare finding in the oral cavity. Surgical excision is the treatment of choice and recurrence is not expected.

** Key words:**Benign oral tumor, oral lipoma, lipoma, oral cavity.

## Introduction

Lipoma is a benign mesenchymal tumor composed of mature adipocytes (1,2). They are common in the head and neck region, but their appearance in the oral cavity is uncommon. Only 1-5% of the lesions are located in this area (3,4); representing 2.2% of all lipomas (3). The most common areas are the buccal mucosa, lips, tongue, palate,vestibule, floor of the mouth and retromolar area (5).

Clinically they are well circumscribed, painless and slow growing tumors (6-8). Their etiology and pathogenesis are not clear, even though factors like mechanical, endocrine, inflammatory (6,8-10), hypercholesterolemia and obesity (11,12), radiation (11) as well as chromosomal abnormalities (3,9,13) have been considered. Histologically they can be classified as simple lipoma, fibrolipoma, spindle cell lipoma, intramuscular lipoma, chondrolipoma, pleomorphic lipoma, myxoid lipoma, angiolipoma and sialolipoma (3,6,14).

The most accepted treatment is surgical excision, but medical management has also been proposed (4,5). Recurrence is rare (4).

In this paper we present a review of the literature of the past 10 years and two clinical cases.

## Material and Methods

A literature review of the last 10 years (2004)-(2014)in the Medline_Pubmed database and ScIELO (Scientific Electronic Library Online) was done using the keywords ”*intraoral lipoma OR oral cavity lipoma*”. Selection criteria included literature reviews, case series and case reports in humans; in English and in Spanish. Articles which did not include intraoral lipomas or information about sex, age and/or size and lipomas localized in the parotid gland were eliminated. The papers reviewed specifically analyze the number of cases, sex, age, location, size and histological pattern.

In this review we present two cases of lipoma.

## Results

**Figure 1:**
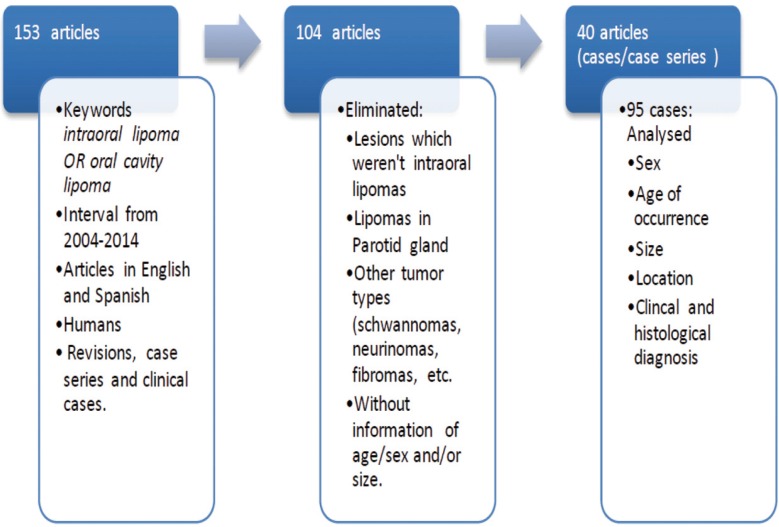
Flow chart with the selection criterion for the inclusion of selected publications.

157 articles were initially found, which after the application of the selection criteria were reduced to 40 case series and clinical case reports which included a total of 95 intraoral lipomas (Fig. 1).  Table 1 summarizes the most significant data obtained from each article (1-40).

**Table 1:**
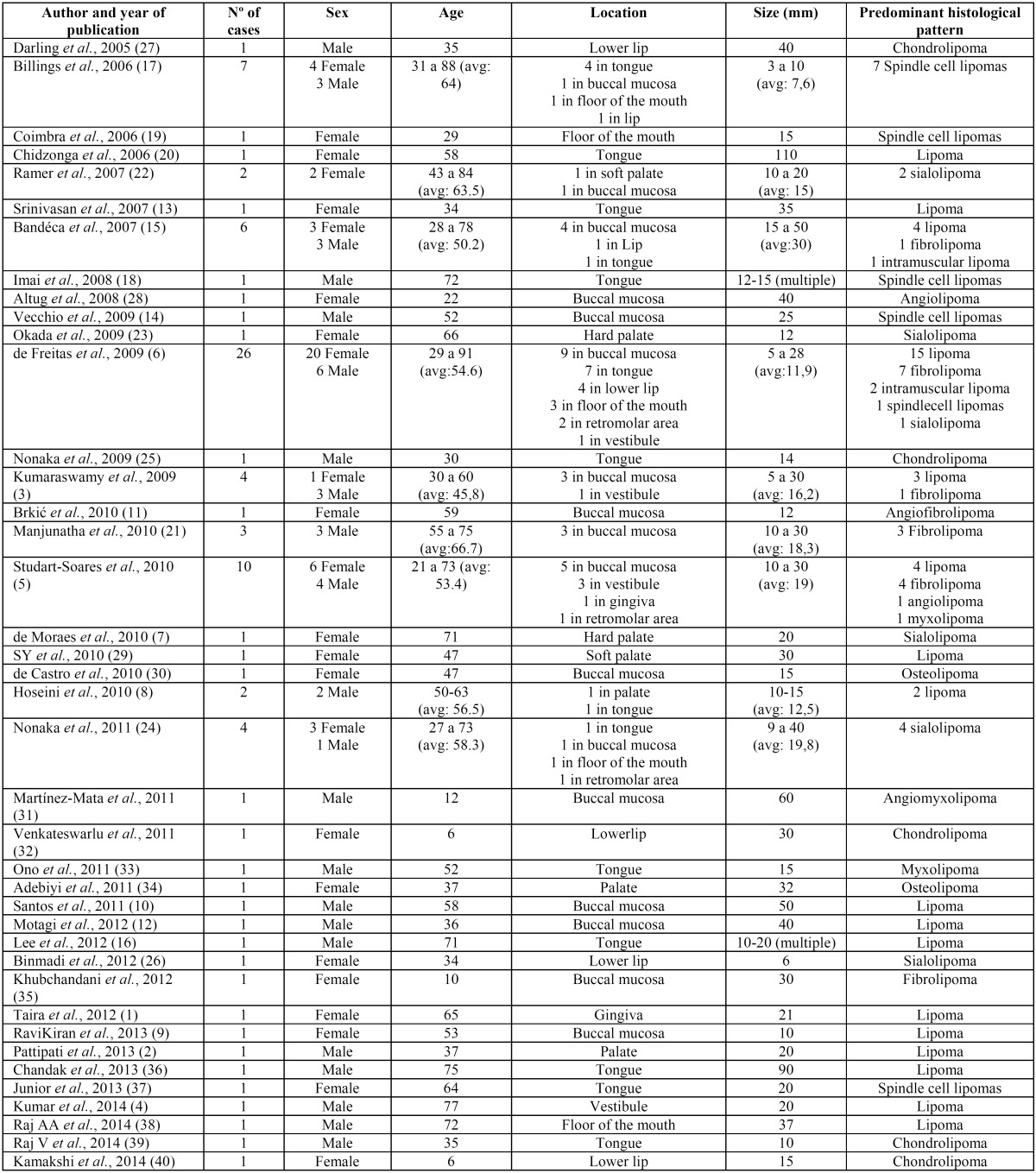
Articles reviewed with their most significant data.

With regards to sex distribution, 56 cases were found in women (58.9%), 39 in men (42.2%). The average age was found to be 52.28 years (52.28 ± 18.55); and it is noteworthy that the majority of lesions occurred in the fourth and sixth decade of life.

The most common region for the occurrence was the buccal mucosa (n=36, 37.9%), followed by the tongue (n=23, 24.2%), lip (n=10, 10.5%), palate (n=7, 7.4%), floor of the mouth (n=7, 7.4%), vestibule (n=6, 6.3%), retromolar area (n=4, 4.2%) and gingiva (n = 2, 2.1%) ( Table 2).

**Table 2:**
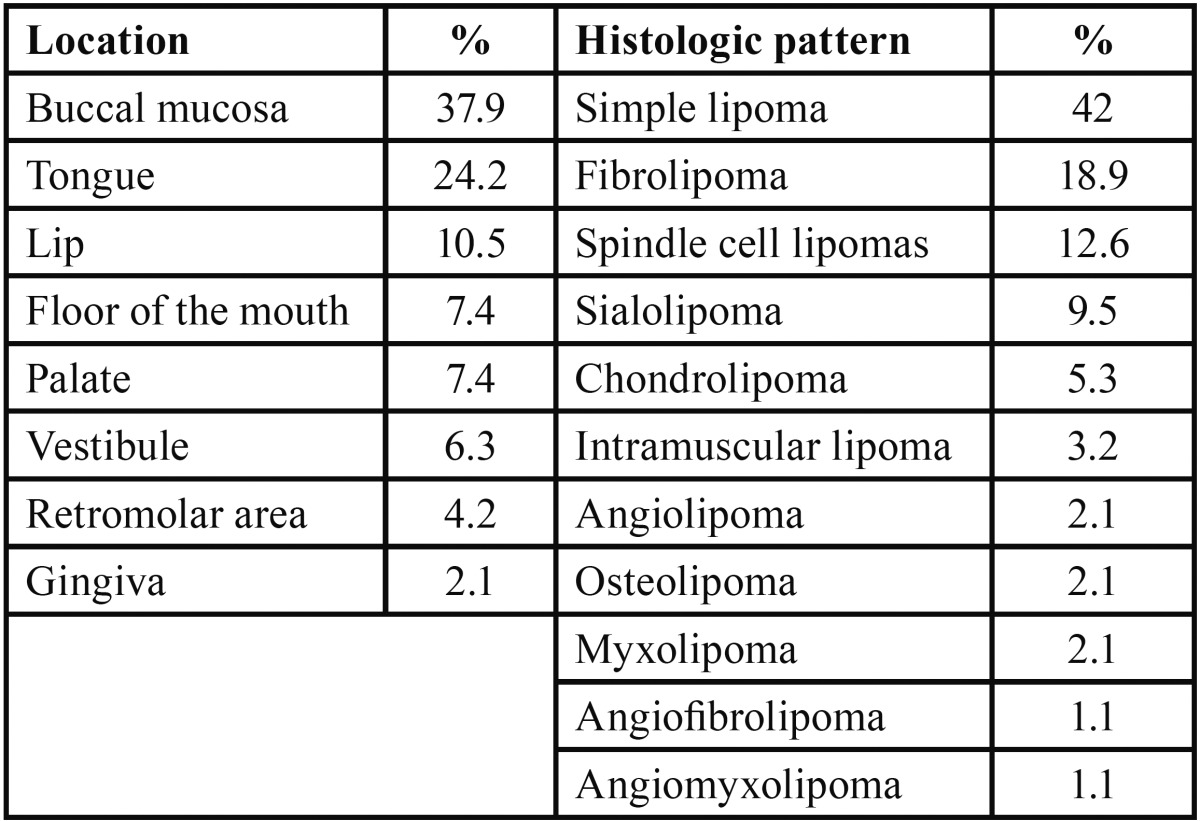
Location and histologic pattern.

Regarding the histological type, the most common pattern was simple lipomas (n=40, 42%), fibrolipomas (n=18, 18.9%), followed by spindle cell lipoma (n=12, 12.6 %), sialolipomas (n=9, 9.5%), chondrolipomas (n=5, 5.3%), intramuscular lipomas (n=3, 3.2%), angiolipomas (n=2, 2.1% ), osteolipomas (n=2, 2.1%), myxolipomas (n=2, 2.1%), angiofibrolipomas (n=1, 1.1%) and angiomyxolipomas (n=1, 1.1%) ( Table 2).

With reference to the size of lipomas reviewed, the average size was recorded to be 19.77 ± 16.26 mm and two of the lesions were multiple.

-Case report nº 1

**Figure 2:**
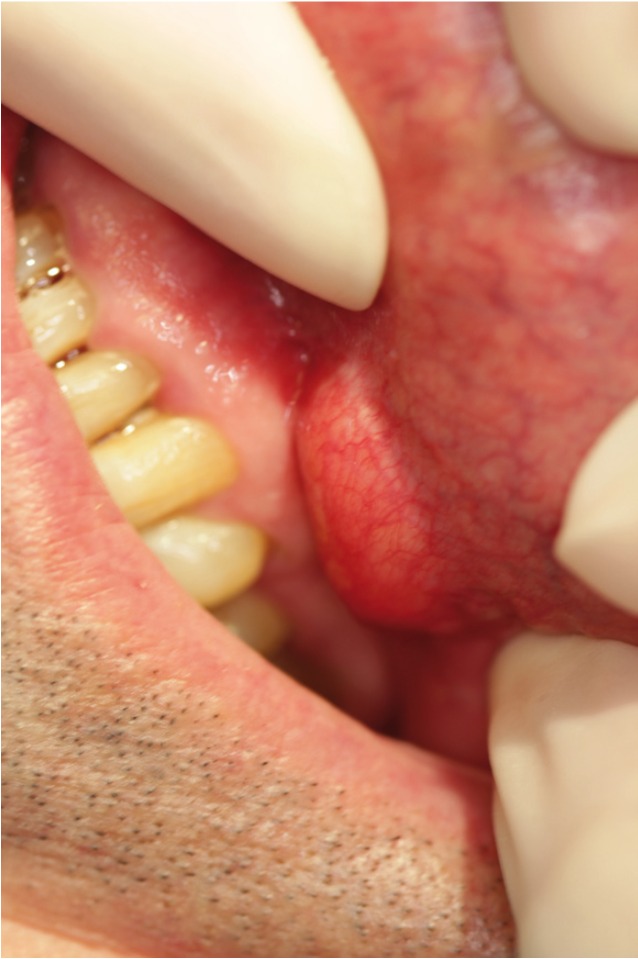
Submucosal mass in the left mandibular vestibule.

We report a case of a 61 year old man with an accidental finding of a lesion; a single painless swelling in the right lower vestibule. Clinical examination revealed a mass of 1.6x1.7cm, soft, mobile, not attached to deeper planes and covered by mucosa which appeared normal but with a slight yellowish color. No neurological defects were demonstrated despite its location near the mental foramen, and cervical lymphadenopathy couldn’t be palpated (Fig. 2).

**Figure 3:**
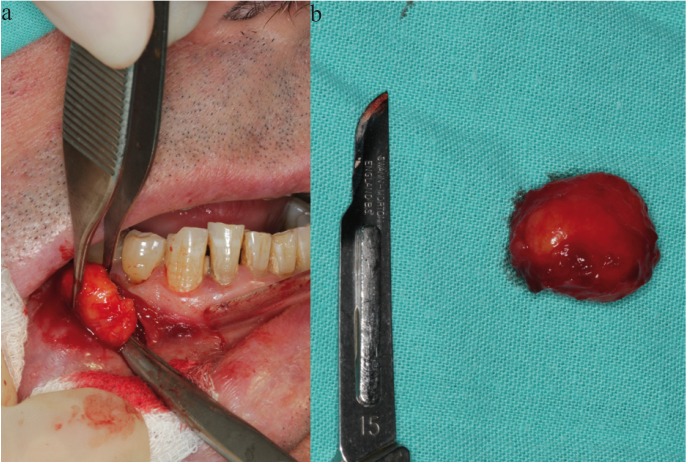
a) Exposing the mass. b) Macroscopic image of the surgically excised tissue.

A differential diagnosis of lipoma was given, which was confirmed with fine needle aspiration cytology (FNAC). The lesion was enucleated under local anesthesia with articaine 1: 100,000 4%, followed by incision, dissection, excision and suture using Vicryl® 3/0. Macroscopically itwas an encapsulated lesion, easily enucleable and yellowish in color (Fig. 3a). Histological examination at 40x magnification (H & E, Hematoxylin & Eosin) showed adipocytes and a well circumscribed tumor with a thin fibrous capsule.

The postoperative period was uneventful and no recurrence was seen after 1 year of follow-up. The definitive diagnosis was confirmed to be lipoma, similar to clinical diagnosis (Fig. 3b).

-Case report nº 2

The second case is of an 89-year-old female with a nodular lesion on the left lingual border with a diameter of 1.2 cm and unknown time of evolution. The lesion was asymptomatic, well defined, smooth with soft consistency and with anormal lingual mucosal surface.

A presumptive diagnosis of fibroma was made. An excisional biopsy and enucleation of the lesion was performed. Macroscopically an encapsulated and yellowish lesion was observed. The definitive diagnosis was lipoma and there was no recurrence.

Histological examination with H & E staining revealed mature fat cells that differed little in microscopic appearance from the surrounding normal fatty tissue. The epithelium was found to be stratified squamous parakeratinized epithelium and fibrocellular connective tissue stroma, having abundant groups of oval cells with vacuolated peripheral nuclei planes, resembling adipocytes, characteristic of lipoma.

## Discussion

While intraoral lipomas are relatively uncommon (1,4,7,8,15,16), their clinical diagnosis is easy due to their yellowish color, and their usual location superficially near the mucosa (1). The differential diagnosis includes fibroma, dermoid cyst, minor salivary gland tumors, mucocele, hemangioma, lymphangioma, rhabdomyoma or neuroma (4,17) ( Table 3). Depending on its location, a herniated buccal fat of pad should also be kept in mind while performing a differential diagnosis (3).

**Table 3:**
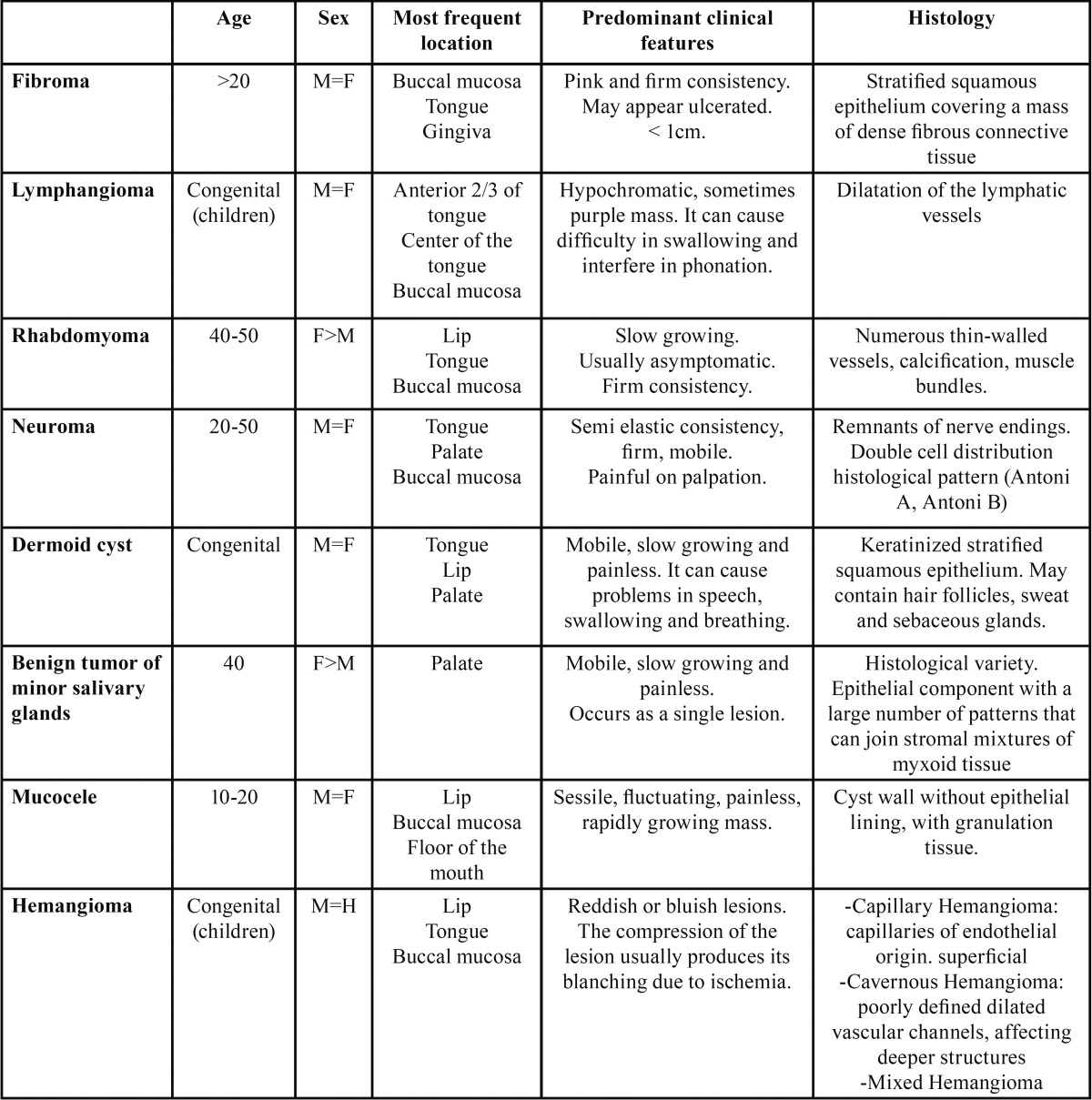
Differential diagnosis of intraoral lipoma.

The majority of cases are solitary tumors. The literature shows that only 5% occur as multiple lesions (4,13,16,18). We found 2 cases of multiple lipomas in our review (16,18). When they occur as multiple lesions they may be associated with syndromes such as neurofibromatosis, Gardner’s syndrome, Dercum’s disease, familial multiple lipomatosis, Proteus syndrome or Pai syndrome (4).

We have already mentioned that the etiology is unknown, but two main theories have been established. (i) The “Hypertrophy theory”, which states that obesity and inadvertent growth of adipose tissue may contribute to their formation. This theory is less convincing at explaining the lesions that occur in areas lacking pre-existing adipose tissue (4). (ii) The “Metaplasia theory” suggests that the lipomatous development occurs due to aberrant differentiation of mesenchymal cells in lipoblasts (3,4,19). Other mechanisms such as trauma, infection, chromosomal abnormalities or hormonal imbalances have also been proposed (3,6,11).

According to the literature,the most common sites of this type of tumor are the buccal mucosa and the tongue (20,21). Studart-Soares *et al.* (5), revised 450 intraoral lipomas between 1966 and 2009, and the most common site was found to be buccal mucosa (n=174; 38.7%), followed by vestibule (n=35; 7.8%), retromolar area (n=21; 4.7%), and other sites (n=220; 48.8%). Taira *et al.* (1), studied 207 cases published between 1987 and 2004, andalso found the buccal mucosa to be the most prominent site (n=84, 40.6%), followed by tongue (n=37, 17.9%), lip (n=26, 12.6%), and other areas (n=60, 28.9%). With respect to the histology, the sialolipoma variety is generally encountered in minor salivary glands (22-24). Our review also found the buccal mucosa to be the most common site for lipoma (n=38; 34.9%), in accordance with the literature.

With respect to the sex distribution in 450 cases studied by Studart-Soares *et al.* (5), 256 were males (52.2%) and 234 were females (47.8%). The tendency was similar in the review conducted by Taira *et al.* (1). On the other hand, in the study of 26 cases by Freitas *et al.* (6), 20 were females (76.92%) and 6 were males (23.8%). In our revision, we found a female predilection with 56 cases in females (58.9%), as compared to 39 males (41.1%). Thus, if we rely on the literature, there is practically no difference in distribution between the sexes, with a male to female ratio of 1:1.2 (4). With respect to the age of distribution, all the articles we studied seemed to coincide with our revision, with the majority of the lipoma cases occurring between the 4th and 6th decade of life (1,5).

Histologically, lipomas are classified based on the matrix and the properties of tumor cells: Simple lipoma, fibrolipoma, spindle cell lipoma, intramuscular lipoma, angiolipoma, chondrolipoma, pleomorphic lipoma, myxoid lipoma and sialolipoma (3,17,25,26). Studart-Soares *et al.* (5) investigated the histological type of 390 cases, in which the most common histologic pattern was simple lipoma (n=229, 48.7%), followed by fibrolipomas (n=103, 26.4%), myxolipoma (n=9, 2.3%), angiolipomas (n=4, 1%) and others (n=45, 11.5%). Taira *et al.* (1), analyzed the histological pattern of 113 cases in their study; and the prevalence of various histological types in descending order were simple lipomas (n=78, 69%), fibrolipomas (n=31, 27.4%) and others (n=4, 3.5%). This trend coincides with the one we found in this review; we found predominance of simple lipomas (n=40, 42%), followed by fibrolipomas (n=18, 18.9%), spindle cell lipomas (n=12, 12.6%) and finally other histological types (n = 25, 26.3%).

The size of lipomas varies greatly, although most of the lesions are less than 10mm (3), reaching up to 11cm in diameter (20). In this review, the average size was 19.77mm, the largest recorded lesion being 110 mm.

The treatment of choice is surgical excision. No recurrence has been described, although it may occur in the case of infiltrating lipomas basically due to an inadequate excision combined with a non-encapsulated lesion. Malignant transformation hasn’t been described either (4). Medical management of lipomas has also been proposed which involves injecting steroids to cause atrophy of adipose tissue. Lesions which are less than 2.5cm in diameter show a better prognosis. The injection of a mixture of 1:1 parts lidocaine with triamcinolone acetonideis repeated once a month. The average volume used ranges from 1 to 3 ml depending on the size of the tumor. Liposuction is also used using a 16-gauge needle in average (4 to 10 cm) or large-sized (> 10cm) tumors (4). In this review all lipomas were treated by surgical excision and none of them showed any recurrence.
